# A Systematic Review of Risk Factors Associated with Surgical Site Infections among Surgical Patients

**DOI:** 10.1371/journal.pone.0083743

**Published:** 2013-12-18

**Authors:** Ellen Korol, Karissa Johnston, Nathalie Waser, Frangiscos Sifakis, Hasan S. Jafri, Mathew Lo, Moe H. Kyaw

**Affiliations:** 1 Oxford Outcomes, Vancouver, British Columbia, Canada; 2 MedImmune, Gaithersburg, Maryland, United States of America; Aligarh Muslim University, India

## Abstract

**Importance:**

Surgical site infection (SSI) complicates 2-5% of surgeries in the United States. Severity of SSI ranges from superficial skin infection to life-threatening conditions such as severe sepsis, and SSIs are responsible for increased morbidity, mortality, and economic burden associated with surgery. *Staphylococcus aureus* (*S. aureus*) is a commonly-isolated organism for SSI, and methicillin-resistant *S. aureus* SSI incidence is increasing globally.

**Objective:**

The objective of this systematic review was to characterize risk factors for SSI within observational studies describing incidence of SSI in a real-world setting.

**Evidence Review:**

An initial search identified 328 titles published in 2002-2012; 57 were identified as relevant for data extraction. Extracted information included study design and methodology, reported cumulative incidence and post-surgical time until onset of SSI, and odds ratios and associated variability for all factors considered in univariate and/or multivariable analyses.

**Findings:**

Median SSI incidence was 3.7%, ranging from 0.1% to 50.4%. Incidence of overall SSI and *S. aureus* SSI were both highest in tumor-related and transplant surgeries. Median time until SSI onset was 17.0 days, with longer time-to-onset for orthopedic and transplant surgeries. Risk factors consistently identified as associated with SSI included co-morbidities, advanced age, risk indices, patient frailty, and surgery complexity. Thirteen studies considered diabetes as a risk factor in multivariable analysis; 85% found a significant association with SSI, with odds ratios ranging from 1.5-24.3. Longer surgeries were associated with increased SSI risk, with a median odds ratio of 2.3 across 11 studies reporting significant results.

**Conclusions and Relevance:**

In a broad review of published literature, risk factors for SSI were characterized as describing reduced fitness, patient frailty, surgery duration, and complexity. Recognition of risk factors frequently associated with SSI allows for identification of such patients with the greatest need for optimal preventive measures to be identified and pre-treatment prior to surgery.

## Introduction

Surgical site infection (SSI) is a commonly-occurring healthcare-associated infection, complicating 2-5% of surgeries in the United States (US)[1]. Increased morbidity and mortality are associated with SSI, ranging from wound discharge associated with superficial skin infection to life-threatening conditions such as severe sepsis[1,2]. SSIs are responsible for an increased economic burden to healthcare systems, including additional postoperative hospital duration and costs[1]. *Staphylococcus aureus* is a commonly-isolated organism in SSI, accounting for 15-20% of SSI occurring in hospital; other organisms regularly isolated from SSIs include gram-negative bacilli, coagulase-negative staphylococci, *Enterococcus* spp., and *Escherichia coli*[1–3]. Methicillin-resistant *S. aureus* (MRSA) is an increasingly important pathogen that causes more than 50% of *S. aureus* hospital-acquired infections in the US and Europe, and presents challenges to treatment due to multiple antibiotic resistance[4,5]. 

The risk of developing an SSI is multifactorial. In observational studies, a wider breadth of risk factors and their impact on incidence of SSI can be observed based on routine clinical practice, and for a larger range of patients, as opposed to the narrow focus on particular risk factors that may be considered within clinical trials. However, investigators of observational studies cannot control the specific variables and level of detail available and it can be challenging to comprehensively adjust for all relevant confounding variables in the estimation of particular risk factors for SSI[6]. To date, overarching syntheses of the data available regarding risk factors for SSI in real world settings has been limited.

As SSIs continue to pose challenges in healthcare management, detailed and specific identification of the factors that may place individual patients at greater risk of infection, and identification of the gaps in currently-available prevention options could help to minimize morbidity, mortality and healthcare costs associated with SSI. The objective of this systematic literature review was to describe the frequency of and factors associated with SSI, *S. aureus* SSI, and MRSA SSI in a real-world observational setting, as they have been published in the medical, peer-reviewed literature. As the studies included in this review were observational in nature, risk factors were observed in a real-world setting rather than a randomized controlled trial. The potential for confounding and other sources of bias to have influenced observed results was considered and discussed. 

## Methods

### Search Strategy and Selection Criteria

The methodology for this systematic review was based on the Preferred Reporting Items for Systematic Reviews (PRISMA) reporting guidelines[7]. Due to the focus of PRISMA guidelines on systematic reviews reporting randomized trial or interventional studies, not all guidelines were relevant to this review of observational studies.

Literature was searched from MEDLINE, EMBASE, the Database of Abstracts of Reviews of Effects and the Cochrane Database of Systematic Reviews. The search strategy was limited to articles published in the English language between 1 January 2002 and 31 May, 2012. This search was supplemented by a PubMed search conducted on 31 May 2012 in order to include the most recently published articles indexed within MEDLINE. The search strategy required the broad key terms “Surgical site infection,” “Staphylococcus aureus”, and “Risk factor.” Article titles, abstracts and full-texts were assessed by two independent reviewers against established inclusion criteria; discrepancies between the reviewers were resolved through consensus. Criteria for inclusion, which were applied at all review stages, required that studies: (1) be observational and published in a peer-reviewed journal, (2) report a relative effect for a risk factor of SSI post-surgery; and (3) discuss *S. aureus* infections, including but not limited to MRSA. Reference lists of included articles were searched for additional relevant sources. Potential eligibility based on inclusion criteria was assessed in a title review, followed by an abstract review. Articles for which the abstract review suggested potential eligibility were assessed in full-text. For articles that were excluded at any stage, the specific reason for exclusion was documented. 

Extracted data included study design, institutional factors, baseline population and operative characteristics, incidence of SSI (*S. aureus*, MRSA, superficial incisional-, deep incisional-, and organ-space), time until onset of infection, and risk factor estimates including odds ratios, confidence intervals and p-values for statistical significance. When information was unclear or missing from a publication, the authors were contacted. In cases where the corresponding author did not respond after multiple contact attempts, the publication was excluded from analysis.

### Evidence Synthesis

Study design characteristics and risk factors were summarized as counts and percentages. Within each study, cumulative incidence was calculated for overall SSI, *S. aureus* SSI, and MRSA SSI. Cumulative incidence was calculated using a numerator of all identified infections and a denominator of all surgeries eligible for inclusion throughout the follow-up period of each study; for the majority of studies this follow-up period was 30 days for surgeries not involving an implant and one year for surgeries involving an implant. Incidence calculations included multiple surgeries per person for studies in which individuals were eligible to have more than one included surgery and contribute more than one infection to the total count. 

When reporting measures of association between risk factors and infection outcomes, a large majority of studies (93%) – both retrospective and prospective – reported odds ratios; while a small number of prospective studies reported relative risks, given the relatively low incidence rates of SSI and the relatively small magnitude of most effect sizes reported here, relative risks can be interpreted as approximations of odds ratios[8]; to facilitate synthesis in reporting, all relative effect results were interpreted on the odds ratio scale. 

Odds ratios were characterized by the following measures: the number of regression models across studies in which the risk factor was included, the range of estimates across studies, and, amongst statistically significant estimates (defined as p ≤0.05), the number that were identified as risk factors (i.e. odds ratio >1.0) vs. protective effects (i.e. odds ratio <1.0). A measure of centrality (e.g. mean, median) was not reported across studies due to different variable definitions applied across studies, such as continuous vs. categorical variables or different categorical cutpoints, which prevent numerical estimates from being consistently and meaningfully combined across studies. 

## Results

**Figure 1:**
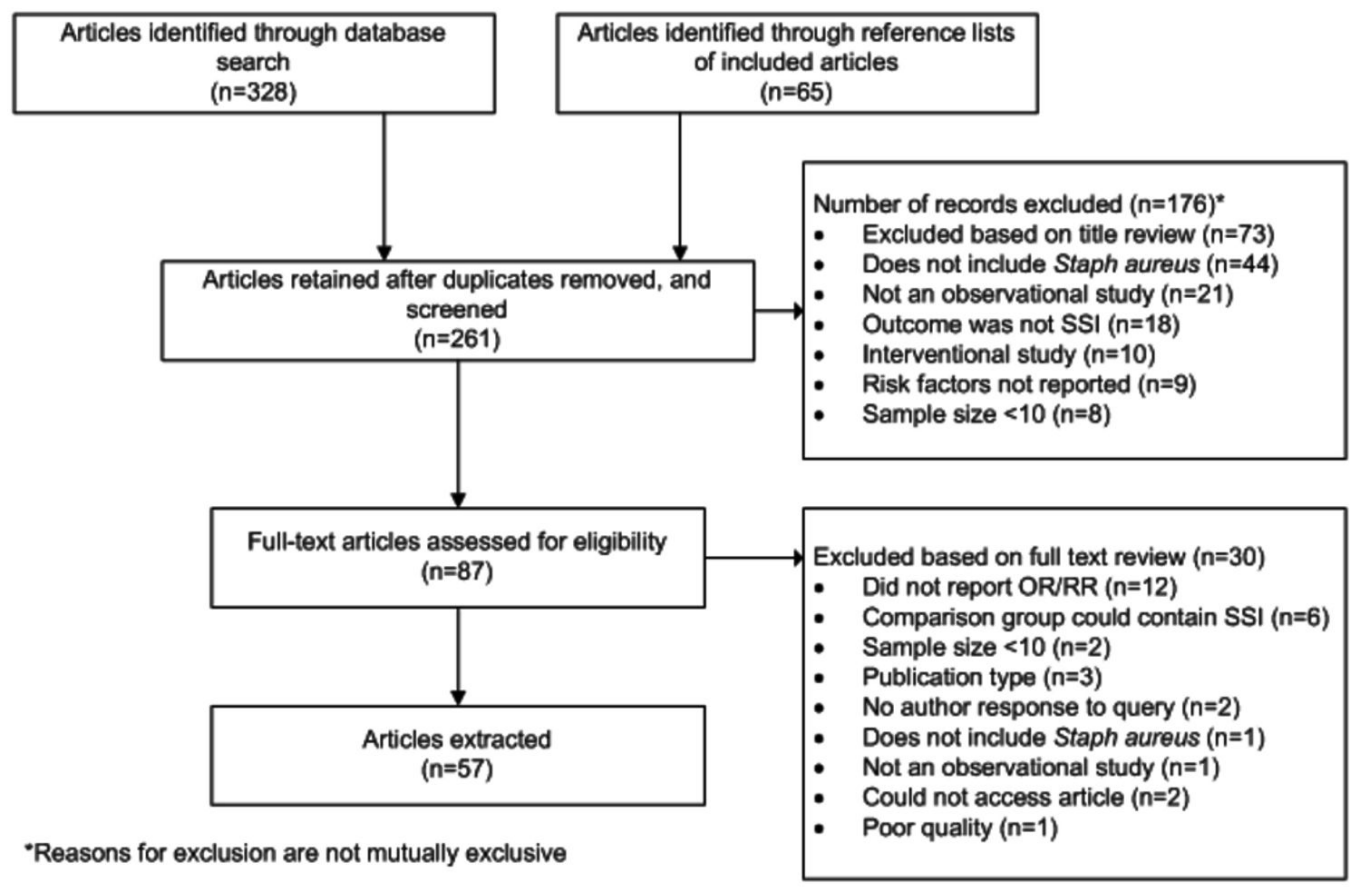
Flow diagram depicting articles excluded from the review, including stage of, and reason for, exclusion in systematic review of risk factors for surgical site infection.

A summary of the number of titles, abstracts, and full-text articles reviewed, and reasons for exclusion are presented in Figure 1. In addition to following the inclusion criteria set *a priori*, four studies were excluded because of concerns that individuals with SSI may have been included in the comparison group. The findings presenting the key elements of each study included in the systematic review are summarized in Appendix Table S1.

Within the 57 studies identified for extraction, a number of studies included multiple analyses. For the incidence analysis, 60 unique numerator and denominator estimates were identified across the 57 studies. In the risk factor analysis, the number of models in which each risk factor was included varied across specific risk factors; for each factor, the number of models was recorded and this value served as the denominator for related analyses. 

An overview of key study characteristics is given in Table 1. A more comprehensive list of the frequency of specific risk factors is presented in Appendix Table S2. Approximately 90% of studies utilized either a cohort or case-control design. While individual study designs varied, all studies described a systematic sampling strategy in which all surgeries meeting pre-defined inclusion criteria were considered. There were 25 studies (43.1%) from the US, and 20 studies (35.1%) from Europe or Canada. Four studies were based in Eastern Asia, and eight were from other geographic regions. The median sample size of studies was 437 surgeries, with sample sizes ranging from a prospective cohort of 15 orthopaedic surgeries in Japan[9], to a national surveillance database of over 70,000 surgeries in the Netherlands[10].

**Table 1:** Characteristics of study design in 57 studies meeting full-text inclusion criteria.

	**Studies included**	
	**(N=57)**	
**Characteristic**	**n**	**%**
Study design		
Cohort	31	54.4
Case-control	20	35.1
Chart review	2	3.5
Other	4	7.0
Study perspective^1^		
Prospective	32	56.1
Retrospective	24	42.1
SSI definition		
CDC/NNIS	41	71.9
CDC/NHSN	1	1.8
Not specified	13	26.3
Other	2	3.5
Geographical location		
United States	25	43.9
Europe/Canada^2^	20	35.1
Eastern Asia^3^	4	7.0
Other^4^	8	14.0

Abbreviations: CDC = Centers for Disease Control and Prevention; NHSN = National Healthcare Safety Network; NNIS = Nosocomial Infections Surveillance System; n = number.

^1^ One study did not provide enough information to determine if the study was prospective or retrospective

^2^ Studies from European countries: Cyprus, England, France, Germany, Italy, Serbia, Spain, Switzerland, The Netherlands, Turkey and the United Kingdom.

^3^ Studies from Asian countries: Japan, Korea and Thailand.

^4^ Studies from other countries: Australia, Brazil, Iran, Mexico, New Zealand, Nigeria, Pakistan and Tanzania.

**Figure 2:**
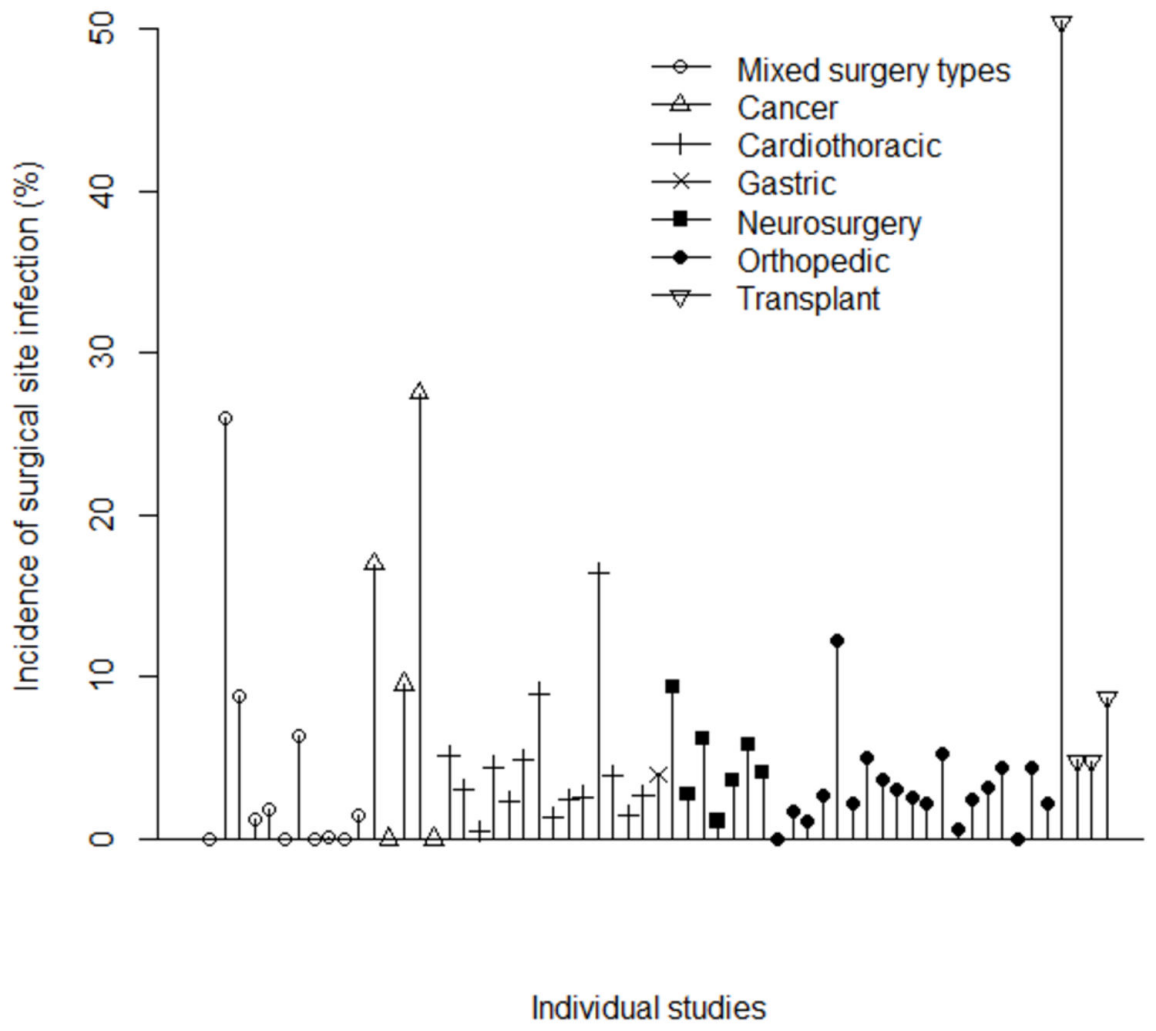
Study-level reported incidence of surgical site infection, stratified by surgery type.

Cumulative incidence of SSI, overall, and stratified by type of surgery are summarized across studies in Table 2 and displayed at the individual-study level in Figure 2. Within the 57 studies, there were 61 unique reports of overall SSI incidence, 55 studies reported *S. aureus* incidence and 39 reported MRSA incidence. The median overall SSI cumulative incidence, across all studies, was 3.7%. Incidence ranged from 0.1% to 50.4%; the incidence of 50.4% was observed in a study describing transplant surgery. The surgery types associated with the highest SSI incidence were tumor-related and transplant surgeries; this was true for SSI, *S. aureus* and MRSA SSI. The median incidence among subgroups of SSI was 2.2% for superficial infections (29 studies), 1.2% for deep incisional infections (31 studies), and 0.6% for organ-space infections (15 studies).

**Table 2:** Incidence of surgical site infections and time until infection onset as reported in 60 analyses performed across 57 studies^1^.

**Surgery type**	**% Incidence of infection: Median (Range)**						**Time to SSI onset (days post-surgery): Median (n=17)**	
	**SSI (n=60)**		***S. aureus* SSI (n=55)**		**MRSA SSI (n=39)**			
Overall (n=61)^1^	3.7	(0.1 - 50.4)	1.8	(0.1 - 56.0)	0.8	(0.0 - 32.0)	17.0	(6.2 - 41.4)
Surgery type								
Mixed surgeries (n=11)	1.9	(0.1 - 26.0)	1.5	(0.1 - 6.4)	0.5	(0.1 - 10.2)	7.2	(6.2 - 8.2)
Cardiothoracic (n=14)	2.8	(0.5 - 16.4)	1.3	(0.3 - 56.0)	0.5	(0.0 - 32.0)	9.9	(9.0 - 17.0)
Neurosurgery (n=7)	4.2	(1.1 - 9.4)	2.3	(0.6 - 5.5)	0.7	(0.1 - 1.1)	15.0	(13.5 - 20.5)
Tumor-related surgery (n=5)	17.0	(9.6 - 27.5)	6.1	(1.9 - 11.9)	1.3	(1.3 - 1.3)	17.9	(17.0 - 34.0)
Orthopedics (n=19)	2.7	(0.6 - 12.2)	1.6	(0.4 - 4.4)	0.8	(0.3 - 2.5)	33.5	(13.5 - 41.4)
Transplant (n=4)	6.8	(4.8 - 50.4)	4.8	(1.0 - 15.0)	6.3	(1.0 - 11.5)	41.0	(41.0 - 41.0)
Gastric (n=1)	4.0	(4.0 - 4.0)	0.5	(0.5 - 0.5)	0.4	(0.4 - 0.4)	8.0	(8.0 - 8.0)

Abbreviations: MRSA = methicillin-resistant *Staphylococcus aureus*; MSSA = methicillin-susceptible *Staphylococcus aureus*; n = number; *S. aureus* = *Staphylococcus aureus*; SSI = surgical site infection.

^1^ Fifty-seven studies were included, however Ridgeway et al.[64] and Gupta et al.[52] reported cumulative incidence multiple analyses.

^2^ Restricted to studies reporting foreign body medical devices that were permanently implanted during surgery.

Seventeen studies reported time until onset of SSI with a median overall time of 17.0 days post-surgery, ranging from 6.2 to 41.4 days. Time until onset tended to be highest in orthopedics and transplant surgeries, potentially due to risk of delayed infection associated with implantation of a foreign object. 

Unadjusted and adjusted odds ratios are presented in Table 3 for key risk factors that were either identified *a priori* as being of particular interest or noted as frequently reported across studies. Variables that were most consistently found to have odds ratios >1 for all infections (i.e. SSI, *S. aureus* SSI and MRSA SSI) in both unadjusted and adjusted analyses included increasing body mass index (BMI), more severe derived risk indices, more severe wound class, diabetes status, and increased surgery duration. Other factors such as increased patient dependence, smoking status, increasing age, *S. aureus* colonization, and use of medical device, were significantly associated with increased risk of all SSIs (i.e. SSI, *S. aureus*, and MRSA SSI) in adjusted analyses. While five studies reported statistically significant unadjusted associations and six studies reported statistically significant adjusted associations between SSI and prophylaxis (Table 3), the majority of these represent comparisons across alternative prophylaxis regimens as opposed to comparison of any prophylaxis use vs. none; one study reported an association between antibiotic prophylaxis and increased odds of SSI[11]; however that report was not corroborated by other studies.

**Table 3:** Odds ratio ranges for estimates of key risk factors for all SSIs, stratified by unadjusted and adjusted methods.

	**n regression models reported**	**Range of estimates**	**n (%) models with statistically significant estimates** ^1^			
**Risk factor**			**Risk factors**		**Protective effect**	
**Unadjusted results**						
Female gender	31	0.4 - 3.5	5	(16.1)	4	(12.9)
Increasing age	21	0.6 - 8.5	9	(42.9)	1	(4.8)
Increasing BMI	23	0.4 - 9.8	12	(52.2)	0	(0.0)
More severe ASA score	19	0.5 - 44.8	12	(63.2)	0	(0.0)
More severe NNIS score	5	0.7 - 4.3	4	(80.0)	0	(0.0)
Diabetes	24	0.7 - 29.6	10	(41.7)	0	(0.0)
Smoking status	11	0.3 - 27.0	2	(18.2)	2	(18.2)
Increased patient dependence	5	0.4 - 6.3	4	(80.0)	1	(20.0)
*S. aureus* colonization	7	0.0 - 15.5	5	(71.4)	1	(14.3)
Increased length of hospital stay	10	1.0 - 12.9	7	(70.0)	0	(0.0)
Use of medical device^3^	4	0.3 - 5.6	1	(25.0)	1	(25.0)
More severe wound class	14	1.0 - 17.4	9	(64.3)	0	(0.0)
Increased surgery duration	19	0.7 - 9.0	12	(63.2)	0	(0.0)
Prophylaxis	16	0.6 - 18.1	5	(31.3)	0	(0.0)
**Adjusted results**							
Female gender	14	0.4 - 3.3	5	(35.7)	2	(14.3)
Increasing age	15	1.0 - 14.0	10	(66.7)	0	(0.0)
Increasing BMI	20	1.0 - 7.1	17	(85.0)	0	(0.0)
More severe ASA score	7	0.7 - 4.2	3	(42.9)	0	(0.0)
More severe NNIS score	5	1.4 - 4.7	3	(60.0)	0	(0.0)
Diabetes	12	1.5 - 24.3	11	(91.7)	0	(0.0)
Smoking status	3	1.2 - 16.8	2	(66.7)	0	(0.0)
Increased patient dependence	4	0.0 - 4.4	3	(75.0)	0	(0.0)
*S. aureus* colonization	7	0.7 - 12.5	5	(71.4)	0	(0.0)
Increased length of hospital stay	5	0.8 - 10.7	5	(100.0)	1	(20.0)
Use of medical device^2^	2	4.0 - 670.4	2	(100.0)	0	(0.0)
More severe wound class	10	1.7 - 10.7	8	(80.0)	0	(0.0)
Increased surgery duration	12	0.1 - 3.2	8	(66.7)	0	(0.0)
Prophylaxis	7	0.4 - 20.5	6	(85.7)	0	(0.0)

Abbreviations: ASA = American Society of Anesthesiologists; BMI = body mass index; ICU = intensive care unit; NNIS = National Nosocomial Infections Surveillance; n = number; OR = odds ratio; *S. aureus* = *Staphylococcus aureus*.

^1^ Statistical significance defined as p ≤0.05

^2^ Restricted to studies reporting foreign body medical devices that are permanently implanted during surgery

**Figure 3:**
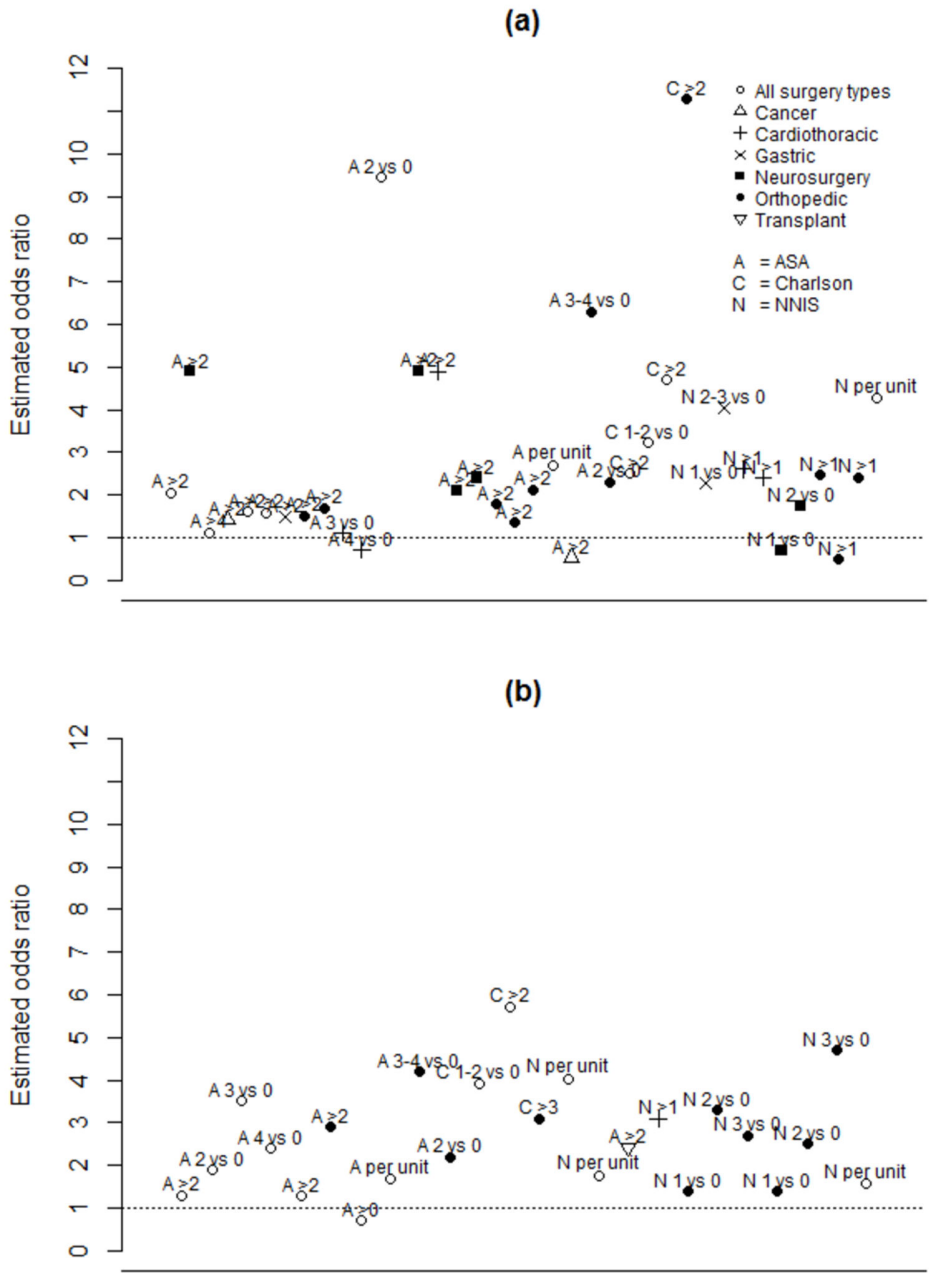
Estimated study-specific odds ratios associated with ASA, Charlson, and NNIS risk scores in (a) unadjusted analyses and (b) adjusted analyses.

Twenty-five studies assessed the relationship between derived risk indices such as the Charlson, National Nosocomial Infections Surveillance (NNIS), or American Society of Anesthesiologists (ASA) indices, and risk of SSI (Figure 3). A number of estimates failed to achieve statistical significance (Table 3), although a large majority of unadjusted and adjusted point estimates indicated a trend towards increased risk. Most estimates were based on a single cutpoint to create a binary indication of high vs. low risk score; however, some estimates were based on the original multi-level scales and indicated a dose-response relationship for the NNIS[12,13] and Charlson[14] indices.

Co-morbidities were consistently found to be associated with SSI incidence. The most frequently considered co-morbidity was diabetes, which was included in 13 adjusted analyses, and 85% of these reported a statistically significant association. Other co-morbidities for which significant adjusted associations were found included chronic obstructive pulmonary disease (COPD)[15–18], coronary heart disease[17], congestive heart failure[19], acute myocardial infarction[20], renal insufficiency[19], hypertension[21] and osteoporosis[17]. The relationship between increasing number of comorbidities and SSI was assessed in several studies. In unadjusted analyses, four studies reported a statistically significant association between increasing number of co-morbidities and SSI[17,22–24], and three studies reported statistically significant adjusted results[22–24]. In adjusted analyses, increasing number of co-morbidities was associated with an estimated odds ratio for SSI of 1.7 (95% CI: 1.3-2.9) per co-morbidity[24], and presence of at least one co-morbidity was associated with an estimated odds ratio for SSI of 2.3 (95% CI: 1.2-4.7)[22] in spinal surgeries and 6.1 (95% CI: 1.3-28.9) in all major surgeries[23].

Ten studies considered risk factors describing patient dependence and frailty, which were characterized in a variety of ways, including independence and activities of daily living[14,15,25–27], incontinence[15,25,28], and admission from a long-term health-care facility[14,27]. The majority of these factors were only considered in unadjusted analyses; adjusted estimates include an odds ratio for SSI of 4.35 (95% CI: 1.64-11.11) associated with admission from a long-term health facility[27], and an odds ratio for SSI of 2.75 (95% CI: 1.16-6.46) associated with requiring assistance with three or more activities of daily living[25].

Variables describing the complexity and/or duration of surgery were also found to be associated with risk of SSI in 16 studies. Duration was defined either relative to a cutpoint (e.g. 75^th^ percentile, 120 minutes, 180 minutes), as a continuous measure per minute of surgery, or as a multi-level categorical variable. Across definitions, increased duration of surgery was consistently found to be associated with increased risk of SSI. When results were restricted to 16 studies that used a binary cutpoint to compare shorter vs. longer surgeries, 15 of 16 estimates suggested an increased risk of SSI for longer surgeries[12,14,15,22,23,25,27,29–36]; 11 of these were statistically significant, with estimated odds ratios ranging from 1.2 to 3.8 with a median value of 2.3. 

Pre-operative length of stay was identified as a significantly associated risk factor for SSI in 12 studies[3,11–14,23,29,33,36–39]. Odds ratios for SSI per additional day of pre-operative stay ranged from 1.0 to 2.0, with a median of 1.1[11,12,14,36–38]. Odds ratios associated with surgeries requiring a prior overnight stay were estimated to be 1.4[15] and 4.6[29]. One study found that pre-operative hospitalizations of up to seven days were not associated with a significant risk of SSI, but that pre-operative stays of eight days or longer were associated with an approximate 10-fold increased risk of SSI[39]. 

## Discussion

In this broad review of the published literature, a number of risk factors for overall SSI, *S. aureus* SSI, and MRSA SSI were identified; these included variables describing reduced patient fitness such as co-morbidities, advanced age, risk indices (ASA or NNIS), increased BMI, and patient dependence. Other important markers included increased length of pre-operative hospital stay, and surgery complexity including increased surgical time. Identified risk factors are biologically plausible, suggesting that patients who are less fit, who have a greater in-hospital exposure time, and/or are undergoing longer and more complex surgeries are at an increased risk for SSI. A statistically significant association between antibiotic prophylaxis and increased risk of SSI observed in one study lacks biologic plausibility as a causal relationship given well-documented evidence regarding a protective effect of antibiotics for SSI[40,41], and increased risks documented in observational studies may be a result of confounding by indication, e.g. due to increased antibiotic use in patients deemed to be at high risk for infection, in more complex surgeries, or in surgeries for which medical errors may have occurred[42,43]. 

As has been noted previously, generating estimates across studies is challenging due to variation in study characteristics, variable definition, specific surgeries included, and study quality[44,45]. As such, overall trends in risk factors were assessed, focusing on direction of effect and achievement of statistical significance, rather than quantitative synthesis across estimates which are not directly comparable. Where applicable, subsets of studies that characterized risk factors using comparable definitions were pooled to generate summary estimates. In addition to variables reported as risk factors for SSI within individual studies, a study-level comparison of reported cumulative incidence (Table 2) provides further insight into surgical-level risk factors, as some studies focused on specific types of surgery. These results suggest that the highest rates of infection are observed in tumor-related surgeries and transplant surgeries; however these are based on observed results across relatively small numbers of studies rather than formal statistical comparisons, and can be interpreted only as exploratory evidence.

Despite widespread adoption of preventive measures by institutions, SSIs continue to occur, and, while the results presented here do not call into question recommendations for existing prevention options, they do suggest a remaining gap and a potential benefit of additional options to further reduce SSI incidence in high-risk patient subgroups. Given that specific patient-level and operative-level risk factors have been consistently observed across studies, and the availability of formal risk indices such as ASA and NNIS scores for identifying high-risk patients, those patients with the greatest need for optimal preventive measures can be identified prior to surgery.

Strengths of this review include the comprehensive nature of study eligibility and risk factor consideration. All observational studies reporting risk factors for SSI across all types of surgery were considered for inclusion, and all risk factor estimates were extracted from each study, giving a broad view of risk factors as observed in routine clinical practice across a variety of settings. Given the variation in studies, a number of stratified analyses were performed to compare results against specific study characteristics, including surgery type, geography, and population characteristics; however, broad trends remained consistent in these stratified analyses and further interpretation was limited due to small study-numbers; these results are not included here. The comprehensive nature of the review also led to limitations; a broad collection of studies with variability in methodology and risk factors considered were included in the review, which presented challenges in numeric synthesis of results. As such, results are primarily focused on the direction of effect, as opposed to magnitude. A more narrow focus on specific risk factors would allow for more detailed exploration of individual trends and magnitude of effect across studies. Results were presented to summarize the entire range of studies, and differences in sample sizes were not accounted for in the synthesis of results.

While the variability across studies limited the ability to generate a single quantitative estimate for specific risk factors, it also provides strength in evidence of the direction of effect for factors such as co-morbidity burden, patient dependence and frailty, and duration and complexity of surgery, which were consistently found to be associated with an increased risk of SSI, across a variety of study designs, study settings, and variable categorizations and definitions. 

## Supporting Information

Table S1 **Characteristics of the 57 studies included.**
(DOCX)

Table S2 **Summary of patient-level, operative, and institutional risk factors considered in unadjusted and adjusted analyses of surgical site infection.**
(DOCX)

Table S3 **Search strategy.**
(DOCX)
