# Supplementary material for: A Systematic Review of Risk Factors Associated with Surgical Site Infections among Surgical Patients
Source: PLoS One. 2013 Dec 18;8(12):e83743. doi: 10.1371/journal.pone.0083743 (PMC3867498; doi:10.1371/journal.pone.0083743)
Supplement: Table S1 — Characteristics of the 57 studies included. (DOCX) [file pone.0083743.s001.docx]

Appendix Table S1: Characteristics of the 57 studies included.

| **Author** | **Country** | **Surgery type** | **Study population** | **Presence of Medical device^1^** | **Definition** | **Outcome** |
| --- | --- | --- | --- | --- | --- | --- |
| Ahmed *et al*. 2011[1] | Pakistan | Cardiothoracic | All | na | CDC/NNIS | SSI & all infections |
| Allpress *et al.* 2004[2] | US | Cardiothoracic | Pediatric | na | CDC/NNIS | SSI |
| Anderson *et al*. 2008[3] | US | Mixed types | All | na | CDC/NNIS | MRSA SSI |
| Apisarnthanarak *et al*. 2003[4] | US | Neurosurgery | All | na | CDC/NNIS | SSI |
| Asensio *et al*. 2008[5] | Spain | Transplant | All | na | CDC^4^ | SSI |
| Blam *et al.* 2003[6] | US | Neurosurgery | All | na | CDC/NNIS | SSI |
| Centofanti *et al*. 2007[7] | Italy | Cardiothoracic | All | na | CDC/NNIS^5^ | SSI |
| Chemaly *et al*. 2010[8] | US | Cancer | All | na | CDC/NNIS | MRSA SSI |
| Chen *et al*. 2010[9] | US | Mixed types | ≥65 y | na | CDC/NNIS | MRSA SSI |
| Chiang *et al*. 2011[10] | US | Neurosurgery | All | 79% | CDC/NNIS | SSI |
| Diez *et al*. 2007[11] | Germany | Cardiothoracic | All | na | na | Mediastinitis |
| Edwards *et al*. 2008[12] | UK | Orthopedic | All | 100% | na | SSI |
| Erman *et al.* 2005[13] | Turkey | Neurosurgery | All | 65% | na^3^ | SSI |
| Fang *et al*. 2005[14] | US | Orthopedic | All | na | na | DWI |
| Felippe *et al*. 2007[15] | Brazil | Cancer | Adults | na | CDC/NNIS | SSI |
| Florescu *et al.* 2012[16] | US | Transplant | All | na | na | *S. aureus* SSI |
| Friedman *et al*. 2007[17] | US | Orthopedic | All | na | CDC/NNIS | SSI |
| Gualis *et al*. 2009[18] | Spain | Cardiothoracic | Adult | na | CDC/NNIS | Mediastinitis |
| Gummert *et al*. 2002[19] | Germany | Cardiothoracic | Adult | na | CDC/NNIS | Mediastinitis |
| Gupta *et al*. 2011[20] | US | Mixed types | Adults | na | na | SSI & MRSA infection |
| Harbarth *et al*. 2008[21] | Swizerland | Mixed types | All | na | CDC/NNIS | MRSA bacteremia secondary to SSI |
| Harrington *et al*. 2004[22] | Australia | Cardiothoracic | All | na | CDC/NNIS | SSI & deep incisional sternal SSI |
| Hass *et al*. 2005[23] | US | Cardiothoracic | All | na | CDC/NNIS | SSI |
| Iinuma *et al.* 2004[24] | Japan | Transplant | All | na | CDC/NNIS | SSI & SSI with secondary bacteremia |
| Kasatpibal *et al*. 2005[25] | Thailand | Mixed types | All | na | CDC/NNIS | SSI |
| Kaya *et al*. 2006[26] | Turkey | Mixed types | All | na | CDC/NNIS | SSI |
| Kaye *et al*. 2006[27] | US | Mixed types | ≥65 y | na | CDC/NNIS | SSI |
| Kaye *et al*. 2009[28] | US | Mixed types | ≥65 y | na | CDC/NNIS | SSI |
| Kim *et al*. 2012[29] | Korea | Gastric | All | na | CDC/NNIS | SSI |
| Koutsoumbelis *et al*. 2011[30] | US | Orthopedic | Adults | na | CDC/NNIS | SSI |
| Kritsotakis *et al*. 2010[31] | Cyprus | Orthopedic | na | 41% | na | SSI |
| Lee *et al*. 2006[32] | US | Orthopedic | All | na | CDC/NNIS | SSI |
| Lepelletier *et al.* 2005[33] | France | Cardiothoracic | All | 49% | CDC/NNIS | Superficial SSI & Mediastinitis & SSI |
| Lietard *et al*. 2008[34] | France | Neurosurgery | ≥16 y | na | CDC/NNIS | SSI |
| Linam *et al*. 2009[35] | US | Orthopedic | Pediatric | na | CDC/NNIS | SSI |
| Marschall *et al*. 2007[36] | US | Cardiothoracic | All | 100% | CDC/NNIS | SSI |
| Mawalla *et al*. 2011[37] | Tanzania | Mixed types | All | na | CDC/NNIS | SSI |
| Minnema *et al*. 2004[38] | Canada | Orthopedic | All | na | CDC/NNIS | SSI |
| Muilwijk *et al*. 2006[39] | The Netherlands | Orthopedic | All | na | CDC/NNIS | Deep & superficial SSI |
| Olsen *et al*. 2003a[40] | US | Cardiothoracic | All | na | CDC/NNIS | Harvest SSI |
| Olsen *et al*. 2003b[41] | US | Neurosurgery | All | na | CDC/NNIS | SSI |
| Omeis *et al*. 2011[42] | US | Cancer | All | na | CDC/NNIS | SSI |
| Omran *et al*. 2007[43] | Iran | Cardiothoracic | All | na | CDC^2^ | DSWI |
| Ramos *et al*. 2008[44] | Spain | Transplant | All | na | CDC/NNIS | Incisional SSI |
| Rao *et al*. 2011[45] | US | Neurosurgery | All | na | CDC/NHSN | Deep SSI |
| Ridgeway *et al*. 2005[46] | UK | Orthopedic | All | 100% | na | SSI |
| Schimmel *et al*. 2010[47] | The Netherlands | Orthopedic | All | na | CDC | Deep SSI |
| Schwarzkopf *et al*. 2011[48] | US | Orthopedic | All | 100% | na | SSI |
| Sciubba *et al*. 2008[49] | Canada | Cancer | All | na | CDC^2^ | SSI |
| Sharma *et al*. 2009[50] | US | Cardiothoracic | All | na | CDC/NNIS | Harvest SSI |
| Shukla *et al*. 2009[51] | UK | Orthopedic | ≥16 y | na | na | MRSA SSI |
| Suljagic *et al*. 2010[52] | Serbia | Mixed types | All | na | CDC/NNIS | SSI |
| Suzuki *et al*. 2010[53] | US | Orthopedic | All | 100% | CDC/NNIS | SSI |
| Thanni *et al*. 2004[54] | Nigeria | Orthopedic | All | 100% | na | SSI |
| Upton *et al*. 2005[55] | New Zealand | Cardiothoracic | Adults | na | na | Staphylococcal mediastinitis (*S. aureus* & other) |
| Vilar-Compte *et al*. 2004[56] | Mexico | Cancer | Adult | na | CDC/NNIS | SSI |
| Yano *et al*. 2009[57] | Japan | Orthopedic | Adult | na | CDC/NNIS | SSI |

Abbreviations: CDC = Center for Disease Control and Prevention; MRSA = methicillin-resistant *Staphylococcus aureus*; NNIS = National Nosocomial Infection Surveillance; SSI = surgical site infection; US = United States.

^1^Medical devices include implants and pace makers.

^2^Follow up surgery up to six months

^3^Two weeks minimum of follow up

^4^Follow up to 720 days

^5^Follow up to 60 days

Notes: Cardiothoracic surgeries included coronary artery bypass graft and cardiovascular procedures; orthopaedic surgeries included hip fracture, internal fixation of long bone fracture, orthopaedic trauma and knee arthroplasty; neurological procedures included spinal fusion, laminectomy, craniotomy, craniectomy and cranioplasty; cancer or tumor-related surgeries included spinal tumor resection and breast cancer and finally transplantation procedures included liver, small bowel and heart transplantations.

Reference List

1. Ahmed D, Cheema FH, Ahmed YI, Schaefle KJ, Azam SI et al. (2011) Incidence and predictors of infection in patients undergoing primary isolated coronary artery bypass grafting: a report from a tertiary care hospital in a developing country. J Cardiovasc Surg (Torino) 52: 99-104. R37116129 [pii].

2. Allpress AL, Rosenthal GL, Goodrich KM, Lupinetti FM, Zerr DM (2004) Risk factors for surgical site infections after pediatric cardiovascular surgery. Pediatr Infect Dis J 23: 231-234. 00006454-200403000-00011 [pii].

3. Anderson DJ, Chen LF, Schmader KE, Sexton DJ, Choi Y et al. (2008) Poor functional status as a risk factor for surgical site infection due to methicillin-resistant Staphylococcus aureus. Infect Control Hosp Epidemiol 29: 832-839. 10.1086/590124 [doi].

4. Apisarnthanarak A, Jones M, Waterman BM, Carroll CM, Bernardi R et al. (2003) Risk factors for spinal surgical-site infections in a community hospital: a case-control study. Infect Control Hosp Epidemiol 24: 31-36. ICHE4794 [pii];10.1086/502112 [doi].

5. Asensio A, Ramos A, Cuervas-Mons V, Cordero E, Sanchez-Turrion V et al. (2008) Effect of antibiotic prophylaxis on the risk of surgical site infection in orthotopic liver transplant. Liver Transpl 14: 799-805. 10.1002/lt.21435 [doi].

6. Blam OG, Vaccaro AR, Vanichkachorn JS, Albert TJ, Hilibrand AS et al. (2003) Risk factors for surgical site infection in the patient with spinal injury. Spine (Phila Pa 1976 ) 28: 1475-1480. 10.1097/01.BRS.0000067109.23914.0A [doi].

7. Centofanti P, Savia F, La TM, Ceresa F, Sansone F et al. (2007) A prospective study of prevalence of 60-days postoperative wound infections after cardiac surgery. An updated risk factor analysis. J Cardiovasc Surg (Torino) 48: 641-646.

8. Chemaly RF, Hachem RY, Husni RN, Bahna B, Abou RG et al. (2010) Characteristics and outcomes of methicillin-resistant Staphylococcus aureus surgical-site infections in patients with cancer: a case-control study. Ann Surg Oncol 17: 1499-1506. 10.1245/s10434-010-0923-5 [doi].

9. Chen TY, Anderson DJ, Chopra T, Choi Y, Schmader KE et al. (2010) Poor functional status is an independent predictor of surgical site infections due to methicillin-resistant Staphylococcus aureus in older adults. J Am Geriatr Soc 58: 527-532. JGS2719 [pii];10.1111/j.1532-5415.2010.02719.x [doi].

10. Chiang HY, Steelman VM, Pottinger JM, Schlueter AJ, Diekema DJ et al. (2011) Clinical significance of positive cranial bone flap cultures and associated risk of surgical site infection after craniotomies or craniectomies. J Neurosurg 114: 1746-1754. 10.3171/2011.1.JNS10782 [doi].

11. Diez C, Koch D, Kuss O, Silber RE, Friedrich I et al. (2007) Risk factors for mediastinitis after cardiac surgery - a retrospective analysis of 1700 patients. J Cardiothorac Surg 2: 23-31. 1749-8090-2-23 [pii];10.1186/1749-8090-2-23 [doi].

12. Edwards C, Counsell A, Boulton C, Moran CG (2008) Early infection after hip fracture surgery: risk factors, costs and outcome. J Bone Joint Surg Br 90: 770-777. 90-B/6/770 [pii];10.1302/0301-620X.90B6.20194 [doi].

13. Erman T, Demirhindi H, Gocer AI, Tuna M, Ildan F et al. (2005) Risk factors for surgical site infections in neurosurgery patients with antibiotic prophylaxis. Surg Neurol 63: 107-112. S0090-3019(04)00521-X [pii];10.1016/j.surneu.2004.04.024 [doi].

14. Fang A, Hu SS, Endres N, Bradford DS (2005) Risk factors for infection after spinal surgery. Spine (Phila Pa 1976 ) 30: 1460-1465. 00007632-200506150-00021 [pii].

15. Felippe WA, Werneck GL, Santoro-Lopes G (2007) Surgical site infection among women discharged with a drain in situ after breast cancer surgery. World J Surg 31: 2293-2299. 10.1007/s00268-007-9248-3 [doi].

16. Florescu DF, Qiu F, Vivekanandan R, Mercer DF, Langnas AN et al. (2012) Risk factors and outcomes of Staphylococcus aureus infections after small bowel and multivisceral transplantation. Pediatr Infect Dis J 31: 25-29. 10.1097/INF.0b013e3182310fb6 [doi].

17. Friedman ND, Sexton DJ, Connelly SM, Kaye KS (2007) Risk factors for surgical site infection complicating laminectomy. Infect Control Hosp Epidemiol 28: 1060-1065. ICHE2006401 [pii];10.1086/519864 [doi].

18. Gualis J, Florez S, Tamayo E, Alvarez FJ, Castrodeza J et al. (2009) Risk factors for mediastinitis and endocarditis after cardiac surgery. Asian Cardiovasc Thorac Ann 17: 612-616. 17/6/612 [pii];10.1177/0218492309349071 [doi].

19. Gummert JF, Barten MJ, Hans C, Kluge M, Doll N et al. (2002) Mediastinitis and cardiac surgery--an updated risk factor analysis in 10,373 consecutive adult patients. Thorac Cardiovasc Surg 50: 87-91. 10.1055/s-2002-26691 [doi].

20. Gupta K, Strymish J, Abi-Haidar Y, Williams SA, Itani KM (2011) Preoperative nasal methicillin-resistant Staphylococcus aureus status, surgical prophylaxis, and risk-adjusted postoperative outcomes in veterans. Infect Control Hosp Epidemiol 32: 791-796. 10.1086/660362 [doi].

21. Harbarth S, Huttner B, Gervaz P, Fankhauser C, Chraiti MN et al. (2008) Risk factors for methicillin-resistant Staphylococcus aureus surgical site infection. Infect Control Hosp Epidemiol 29: 890-893. 10.1086/590193 [doi].

22. Harrington G, Russo P, Spelman D, Borrell S, Watson K et al. (2004) Surgical-site infection rates and risk factor analysis in coronary artery bypass graft surgery. Infect Control Hosp Epidemiol 25: 472-476. ICHE8316 [pii];10.1086/502424 [doi].

23. Haas JP, Evans AM, Preston KE, Larson EL (2005) Risk factors for surgical site infection after cardiac surgery: the role of endogenous flora. Heart Lung 34: 108-114. S0147956304000615 [pii];10.1016/j.hrtlng.2004.02.004 [doi].

24. Iinuma Y, Senda K, Fujihara N, Saito T, Takakura S et al. (2004) Surgical site infection in living-donor liver transplant recipients: a prospective study. Transplantation 78: 704-709. 00007890-200409150-00012 [pii].

25. Kasatpibal N, Jamulitrat S, Chongsuvivatwong V (2005) Standardized incidence rates of surgical site infection: a multicenter study in Thailand. Am J Infect Control 33: 587-594. S0196-6553(05)00436-0 [pii];10.1016/j.ajic.2004.11.012 [doi].

26. Kaya E, Yetim I, Dervisoglu A, Sunbul M, Bek Y (2006) Risk factors for and effect of a one-year surveillance program on surgical site infection at a university hospital in Turkey. Surg Infect (Larchmt ) 7: 519-526. 10.1089/sur.2006.7.519 [doi].

27. Kaye KS, Sloane R, Sexton DJ, Schmader KA (2006) Risk factors for surgical site infections in older people. J Am Geriatr Soc 54: 391-396. JGS651 [pii];10.1111/j.1532-5415.2005.00651.x [doi].

28. Kaye KS, Anderson DJ, Sloane R, Chen LF, Choi Y et al. (2009) The impact of surgical site infection on older operative patients. J Am Geriatr Soc 57: 46-54. JGS2053 [pii];10.1111/j.1532-5415.2008.02053.x [doi].

29. Kim ES, Kim HB, Song KH, Kim YK, Kim HH et al. (2012) Prospective Nationwide Surveillance of Surgical Site Infections after Gastric Surgery and Risk Factor Analysis in the Korean Nosocomial Infections Surveillance System (KONIS). Infect Control Hosp Epidemiol 33: 572-580. 10.1086/665728 [doi].

30. Koutsoumbelis S, Hughes AP, Girardi FP, Cammisa FP, Jr., Finerty EA et al. (2011) Risk factors for postoperative infection following posterior lumbar instrumented arthrodesis. J Bone Joint Surg Am 93: 1627-1633. 10.2106/JBJS.J.00039 [doi].

31. Kritsotakis EI, Dimitriadis I, Bagatzouni D, Alexandrou M, Zinieri V et al. (2010) Surgical site infections after orthopaedic surgery in Cyprus: incidence, risk factors, microbiology and impact. 20th European Congress of Clinical Microbiology and Infectious Diseases .

32. Lee J, Singletary R, Schmader K, Anderson DJ, Bolognesi M et al. (2006) Surgical site infection in the elderly following orthopaedic surgery. Risk factors and outcomes. J Bone Joint Surg Am 88: 1705-1712. 88/8/1705 [pii];10.2106/JBJS.E.01156 [doi].

33. Lepelletier D, Perron S, Bizouarn P, Caillon J, Drugeon H et al. (2005) Surgical-site infection after cardiac surgery: incidence, microbiology, and risk factors. Infect Control Hosp Epidemiol 26: 466-472. ICHE10428 [pii];10.1086/502569 [doi].

34. Lietard C, Thebaud V, Besson G, Lejeune B (2008) Risk factors for neurosurgical site infections: an 18-month prospective survey. J Neurosurg 109: 729-734. 10.3171/JNS/2008/109/10/0729 [doi].

35. Linam WM, Margolis PA, Staat MA, Britto MT, Hornung R et al. (2009) Risk factors associated with surgical site infection after pediatric posterior spinal fusion procedure. Infect Control Hosp Epidemiol 30: 109-116. 10.1086/593952 [doi].

36. Marschall J, Hopkins-Broyles D, Jones M, Fraser VJ, Warren DK (2007) Case-control study of surgical site infections associated with pacemakers and implantable cardioverter-defibrillators. Infect Control Hosp Epidemiol 28: 1299-1304. ICHE2007121 [pii];10.1086/520744 [doi].

37. Mawalla B, Mshana SE, Chalya PL, Imirzalioglu C, Mahalu W (2011) Predictors of surgical site infections among patients undergoing major surgery at Bugando Medical Centre in Northwestern Tanzania. BMC Surg 11: 21. 1471-2482-11-21 [pii];10.1186/1471-2482-11-21 [doi].

38. Minnema B, Vearncombe M, Augustin A, Gollish J, Simor AE (2004) Risk factors for surgical-site infection following primary total knee arthroplasty. Infect Control Hosp Epidemiol 25: 477-480. ICHE8317 [pii];10.1086/502425 [doi].

39. Muilwijk J, Walenkamp GH, Voss A, Wille JC, van den Hof S (2006) Random effect modelling of patient-related risk factors in orthopaedic procedures: results from the Dutch nosocomial infection surveillance network 'PREZIES'. J Hosp Infect 62: 319-326. S0195-6701(05)00339-7 [pii];10.1016/j.jhin.2005.08.006 [doi].

40. Olsen MA, Sundt TM, Lawton JS, Damiano RJ, Jr., Hopkins-Broyles D et al. (2003) Risk factors for leg harvest surgical site infections after coronary artery bypass graft surgery. J Thorac Cardiovasc Surg 126: 992-999. 10.1016/S0022 [doi];S0022522303002009 [pii].

41. Olsen MA, Mayfield J, Lauryssen C, Polish LB, Jones M et al. (2003) Risk factors for surgical site infection in spinal surgery. J Neurosurg 98: 149-155.

42. Omeis IA, Dhir M, Sciubba DM, Gottfried ON, McGirt MJ et al. (2011) Postoperative surgical site infections in patients undergoing spinal tumor surgery: incidence and risk factors. Spine (Phila Pa 1976 ) 36: 1410-1419. 10.1097/BRS.0b013e3181f48fa9 [doi].

43. Omran AS, Karimi A, Ahmadi SH, Davoodi S, Marzban M (2007) Superficial and deep sternal wound infection after more than 9000 coronary artery bypass graft (CABG): incidence, risk factors and mortality. BMC Infect Dis 7: 112-117.

44. Ramos A, Asensio A, Munez E, Torre-Cisneros J, Blanes M et al. (2008) Incisional surgical infection in heart transplantation. Transpl Infect Dis 10: 298-302. TID316 [pii];10.1111/j.1399-3062.2008.00316.x [doi].

45. Rao SB, Vasquez G, Harrop J, Maltenfort M, Stein N et al. (2011) Risk factors for surgical site infections following spinal fusion procedures: a case-control study. Clin Infect Dis 53: 686-692. cir506 [pii];10.1093/cid/cir506 [doi].

46. Ridgeway S, Wilson J, Charlet A, Kafatos G, Pearson A et al. (2005) Infection of the surgical site after arthroplasty of the hip. J Bone Joint Surg Br 87: 844-850. 87-B/6/844 [pii];10.1302/0301-620X.87B6.15121 [doi].

47. Schimmel JJ, Horsting PP, de KM, Wonders G, van LJ (2010) Risk factors for deep surgical site infections after spinal fusion. Eur Spine J 19: 1711-1719. 10.1007/s00586-010-1421-y [doi].

48. Schwarzkopf R, Russell TA, Shea M, Slover JD (2011) Correlation between nutritional status and Staphylococcus colonization in hip and knee replacement patients. Bull NYU Hosp Jt Dis 69: 308-311.

49. Sciubba DM, Nelson C, Gok B, McGirt MJ, McLoughlin GS et al. (2008) Evaluation of factors associated with postoperative infection following sacral tumor resection. J Neurosurg Spine 9: 593-599. 10.3171/SPI.2008.9.0861 [doi].

50. Sharma M, Fakih MG, Berriel-Cass D, Meisner S, Saravolatz L et al. (2009) Harvest surgical site infection following coronary artery bypass grafting: risk factors, microbiology, and outcomes. Am J Infect Control 37: 653-657. S0196-6553(09)00085-6 [pii];10.1016/j.ajic.2008.12.012 [doi].

51. Shukla S, Nixon M, Acharya M, Korim MT, Pandey R (2009) Incidence of MRSA surgical-site infection in MRSA carriers in an orthopaedic trauma unit. J Bone Joint Surg Br 91: 225-228. 91-B/2/225 [pii];10.1302/0301-620X.91B2.21715 [doi].

52. Suljagic V, Jevtic M, Djordjevic B, Jovelic A (2010) Surgical site infections in a tertiary health care center: prospective cohort study. Surg Today 40: 763-771. 10.1007/s00595-009-4124-4 [doi].

53. Suzuki T, Morgan SJ, Smith WR, Stahel PF, Gillani SA et al. (2010) Postoperative surgical site infection following acetabular fracture fixation. Injury 41: 396-399. S0020-1383(09)00615-9 [pii];10.1016/j.injury.2009.11.005 [doi].

54. Thanni LO, Aigoro NO (2004) Surgical site infection complicating internal fixation of fractures: incidence and risk factors. J Natl Med Assoc 96: 1070-1072.

55. Upton A, Roberts SA, Milsom P, Morris AJ (2005) Staphylococcal post-sternotomy mediastinitis: five year audit. ANZ J Surg 75: 198-203. ANS3371 [pii];10.1111/j.1445-2197.2005.03371.x [doi].

56. Vilar-Compte D, Jacquemin B, Robles-Vidal C, Volkow P (2004) Surgical site infections in breast surgery: case-control study. World J Surg 28: 242-246. 10.1007/s00268-003-7193-3 [doi].

57. Yano K, Minoda Y, Sakawa A, Kuwano Y, Kondo K et al. (2009) Positive nasal culture of methicillin-resistant Staphylococcus aureus (MRSA) is a risk factor for surgical site infection in orthopedics. Acta Orthop 80: 486-490. 913080445 [pii];10.3109/17453670903110675 [doi].
