# Supplementary material for: A Systematic Review of Risk Factors Associated with Surgical Site Infections among Surgical Patients
Source: PLoS One. 2013 Dec 18;8(12):e83743. doi: 10.1371/journal.pone.0083743 (PMC3867498; doi:10.1371/journal.pone.0083743)
Supplement: Table S2 — Summary of patient-level, operative, and institutional risk factors considered in unadjusted and adjusted analyses of surgical site infection. (DOCX) [file pone.0083743.s002.docx]

Appendix Table S2: Summary of patient-level, operative, and institutional risk factors considered in unadjusted and adjusted analyses of surgical site infection.

|  | **Studies included (N=57)** | |
| --- | --- | --- |
| **Patient Level Factors** | **n** | **%** |
| Demographic factors |  |  |
| Gender | 34 | 59.6 |
| Age | 27 | 47.4 |
| BMI | 28 | 49.1 |
| Ethnicity | 7 | 12.3 |
| Socio-economic factors | 5 | 8.8 |
| Living in health-care facility | 3 | 5.3 |
| Patient health |  |  |
| Risk scores |  |  |
| ASA score | 19 | 33.3 |
| Charlson score | 4 | 7.0 |
| NNIS risk index | 6 | 10.5 |
| McCabe score | 3 | 5.3 |
| Other risk score | 5 | 8.8 |
| Any risk score | 27 | 47.4 |
| Comorbidities |  |  |
| Diabetes | 31 | 54.4 |
| Heart conditions | 17 | 29.8 |
| Cancer | 10 | 17.5 |
| COPD | 10 | 17.5 |
| Renal disease | 10 | 17.5 |
| Vascular disease | 8 | 14.0 |
| Arthritis | 3 | 5.3 |
| Mental health | 3 | 5.3 |
| Chronic skin condition | 2 | 3.5 |
| Liver disease | 2 | 3.5 |
| Parkinson's disease | 1 | 1.8 |
| Osteoporosis | 1 | 1.8 |
| Respiratory conditions | 1 | 1.8 |
| Other comorbidities | 6 | 10.5 |
| Smoking status | 15 | 26.3 |
| Immunosuppression regimen | 7 | 12.3 |
| Patient dependence | 6 | 10.5 |
| Alcohol abuse | 1 | 1.8 |
| Sleep apnea | 1 | 1.8 |
| *S. aureus* colonization^1^ | 11 | 19.3 |
| **Institutional Level Characteristics** | **n** | **%** |
| Length of stay |  |  |
| Pre-operation | 10 | 17.5 |
| Post-operation | 2 | 3.5 |
| Total length of stay | 2 | 3.5 |
| NICU/PICU/ICU | 5 | 8.8 |
| Previous surgery | 19 | 33.3 |
| Admission to ICU | 2 | 3.5 |
| Physician experience | 3 | 5.3 |
| Hospital experience | 2 | 3.5 |
| Hospital size | 1 | 1.8 |
| **Operation Level Characteristics** | **n** | **%** |
| Medical device |  |  |
| All^2^ | 19 | 33.3 |
| Implant^3^ | 8 | 14.0 |
| Surgical procedures | 28 | 49.1 |
| Blood transfusions | 11 | 19.3 |
| Blood loss | 5 | 8.8 |
| Duration of surgery | 26 | 45.6 |
| Prophylaxis use | 19 | 33.3 |
| Wound class | 15 | 26.3 |
| Surgery Type |  |  |
| Emergency | 14 | 24.6 |
| Elective | 1 | 1.8 |
| Cardiothoracic | 0 | 0.0 |
| Cancer | 1 | 1.8 |
| Neurosurgery | 0 | 0.0 |
| Orthopedic | 0 | 0.0 |
| Transplant | 1 | 1.8 |
| Vascular | 0 | 0.0 |
| Other surgeries | 3 | 5.3 |
| Pre-admission medication | 7 | 12.3 |
| Number of people in operating room | 3 | 5.3 |
| Hair removal | 4 | 7.0 |
| Skin/site preparation | 3 | 5.3 |

Abbreviations: ASA = American Society of Anesthesiologists; BMI = body mass index; COPD = chronic obstructive pulmonary disease; ICU = intensive care unit; NICU = neonatal intensive care unit; NNIS = Nosocomial Infections Surveillance; n = number; PICU = pediatric intensive care unit; *S. aureus* = staphylococcus aureus.
*^1^S. aureus* colonization includes documented *S. aureus* colonization prior to surgery, and previous infection with *S. aureus.*

^2^Medical device – All includes catheters, drains and other temporary devices used during or after a surgical procedure.

^3^Medical device – Implant includes only medical devices that are permanently implanted during surgery.
