# Supplementary material for: A Systematic Review of Risk Factors Associated with Surgical Site Infections among Surgical Patients
Source: PLoS One. 2013 Dec 18;8(12):e83743. doi: 10.1371/journal.pone.0083743 (PMC3867498; doi:10.1371/journal.pone.0083743)
Supplement: Table S3 — Search strategy. (DOCX) [file pone.0083743.s003.docx]

Appendix Table S3: Search strategy

| **Steps** | **Searches** | **Results** |
| --- | --- | --- |
| 1 | surgical site infection$.mp. [mp=ti, ab, tx, kw, ct, sh, hw, tn, ot, dm, mf, dv, nm, ps, rs, ui] | 6145 |
| 2 | surgical-site infection$.mp. [mp=ti, ot, ab, tx, kw, ct, sh, hw, tn, dm, mf, dv, nm, ps, rs, an, ui] | 6145 |
| 3 | 1 and 2 | 6145 |
| 4 | staphylococcus aureus.mp. [mp=ti, ot, ab, tx, kw, ct, sh, hw, tn, dm, mf, dv, nm, ps, rs, an, ui] | 176890 |
| 5 | 3 and 4 | 1092 |
| 6 | risk factor$.mp. [mp=ti, ot, ab, tx, kw, ct, sh, hw, tn, dm, mf, dv, nm, ps, rs, an, ui] | 1283103 |
| 7 | 5 and 6 | 381 |
| 8 | limit 7 to english language [Limit not valid in CDSR,DARE; records were retained] | 358 |
| 9 | limit 8 to yr="2002 -Current" [Limit not valid in DARE; records were retained] | 327 |
| 10 | remove duplicates from 10 | 214 |
